# Enteric alpha defensins in norm and pathology

**DOI:** 10.1186/1476-0711-11-1

**Published:** 2012-01-11

**Authors:** Nikolai A Lisitsyn, Yulia A Bukurova, Inna G Nikitina, George S Krasnov, Yuri Sykulev, Sergey F Beresten

**Affiliations:** 1 Engelhardt Institute of Molecular Biology, Russian Academy of Sciences, 32 Vavilova St., Moscow 119991, Russian Federation; 2 Department of Microbiology and Immunology and Kimmel Cancer Institute, Thomas Jefferson University, Philadelphia, PA 19107, USA

**Keywords:** Enteric alpha defensins, Paneth cells, innate immunity, IBD, colon cancer

## Abstract

Microbes living in the mammalian gut exist in constant contact with immunity system that prevents infection and maintains homeostasis. Enteric alpha defensins play an important role in regulation of bacterial colonization of the gut, as well as in activation of pro- and anti-inflammatory responses of the adaptive immune system cells in lamina propria. This review summarizes currently available data on functions of mammalian enteric alpha defensins in the immune defense and changes in their secretion in intestinal inflammatory diseases and cancer.

## Introduction

Defensins are short, cysteine-rich, cationic peptides found in vertebrates, invertebrates and plants, which play an important role in innate immunity against bacteria, fungi, protozoa, and viruses [1]. Mammalian defensins are predominantly expressed in epithelial cells of skin, respiratory airways, gastrointestinal and genitourinary tracts, which form physical barriers to external infectious agents [2,3], and also in leukocytes (mostly neutrophils), which kill microbes that have already penetrated the body [4]. Mature defensins contain six cysteine residues (Cys_1-6_) forming three intramolecular disulphide bonds. Depending on the bonds arrangement they are classified into alpha, beta and theta subfamilies. Alpha defensins secreted by leukocytes and intestinal Paneth cells of mammals [5] contain disulfide bridges between 1-6, 2-4, and 3-5 cysteine residues, while beta defensins produced by epithelial cells and leukocytes of most multicellular organisms [6] are distinguished by pairing of cysteine residues 1-5, 2-4, and 3-6 [7]. Members of the rare theta defensin subfamily (circular minidefensins) are expressed only in leukocytes and bone marrow cells of monkeys. They are produced by the head-to-tail ligation of two different C-terminally truncated pro-alpha defensins (demidefensins), each nine amino acids long [8].

The tertiary structure of mature alpha defensins consists of a triple-stranded β-sheet with two β-turns [9]. An amphipathic character of the peptide (i.e., mostly hydrophobic structure with a positively charged hydrophilic part) is essential for the insertion into the microbial membrane and the formation of a pore leading to membrane permeabilization and lysis of the microbe [10]. Initial recognition of numerous microbial targets is a consequence of electrostatic interactions between the defensins arginine residues and the negatively charged phospholipids of the microbial cytoplasmic membrane [2,5]. However, the precise mechanism of target recognition and its putative effectors have not been studied in sufficient detail for all known defensin molecules [11]. The most sensitive targets of enteric alpha defensins are Gram-positive and Gram-negative bacteria [12]. Though viruses usually cannot be cleared by the innate immune system, some defensins are able to suppress replication of human immunodeficiency virus [13].

Since their discovery in rabbit neutrophils in 1966 [14], around fifty alpha defensin genes have been identified in primates, rodents, and equines, most of which haven't been analyzed in sufficient detail. The genes evolve extremely rapidly, so that their copy number in various organisms and even in individual genomes of the same species significantly varies [15]. Six best characterized alpha defensins, which are most abundantly expressed in the human body, include four peptides expressed in neutrophils (DEFA1 through 4, previously referred to as human neutrophil peptides HNP1-4) and two enteric defensins - DEFA5 and 6 (formerly designated as HD5 and 6). DEFA5 and 6 are secreted in the mucous layer by Paneth cells of small intestine and colon and to a smaller extent by cells of female reproductive tract and oropharyngeal mucosa [16,17]. Besides, Paneth cells produce more than a dozen of other antimicrobials, including lysozyme, IgAs, angiogenins, and secreted phospholipase A2 [16].

Twenty-four murine alpha defensin genes are listed in the mouse genome informatics database http://www.informatics.jax.org, six of which (enteric alpha defensins 1 through 6, formerly referred to as cryptdins) have been thoroughly investigated in recent years. Unlike humans, rats, and rabbits genomes, mouse DNA does not contain Defa encoding genes that are expressed in neutrophils [18]. Recently, eleven murine alpha defensin related genes (formerly called cryptdin related sequences) with yet unknown functions have been identified. These genes encode prepro-sequences that are nearly identical to those of alpha defensins, but their mature peptides show no homology to defensins [19].

### Gene expression and peptide processing

Human and murine enteric alpha defensin genes consist of two exons, whereas alpha defensin genes expressed in human neutrophils consist of three exons, two of which (second and third) are homologous to enteric defensins [1]. Search for orthologous sequences in promoters of these genes revealed presence of putative binding sites for transcription factors AP1, OCT1.4, and GCN4 [20,21]. Upstream regions of human *DEFA5 *and murine *Defa4 *genes contain highly conserved *cis*-acting elements as evident from the expression of functionally active human peptide in small intestinal crypts of mice transgenic for human *DEFA5 *minigene construct [22].

Analysis of postnatal changes in concentration of murine enteric defensins in the small intestine of conventional and germ-free mice revealed two patterns of gene expression: gradual increase in production of defensins 1, 3 and 6 and rapid raise in secretion of defensins 2, 4 and 5 in the jejunum (but not in the ileum), which is presumably induced by the presence of luminal bacteria [23]. In adult mice alpha defensins are equally expressed along the small intestine except Defa4, which is more abundant in the ileum as compared to the duodenum [24]. Recently, it has been shown, that in human tropical populations secretion of enteric alpha defensins may decrease up to ten times, as compared to Europeans, due to down-regulation of gene expression in response to infections, inflammatory conditions, and malnutrition [25].

Human enteric alpha defensins are synthesized *in vivo *as precursor proteins, some of which have antimicrobial activity. For example human DEFA5 prepropeptide (aa 1-94), contains signal peptide (aa 1-19) and propeptide (aa 20-94), which is subsequently processed into major mature peptide (aa 63-94), and minor mature peptide (aa 56-94) [26]. The prosequence flanking the mature peptide is necessary for correct intracellular sorting and trafficking of the propeptide into the secretory vesicles, where it is proteolytically processed by trypsin during secretion [27,28]. Conversely, murine defensins are stored in vesicles in a mature form after propeptide processing by matrilysin (also known as matrix metalloproteinase Mmp7) [29]. It has been shown that abrogation of murine alpha defensin processing by targeted disruption of the matrilysin gene increases susceptibility of Mmp7 knockout mice to oral challenges with enteric bacteria, whereas transgenic mice overexpressing human DEFA5 are markedly resistant to orally administered virulent *Salmonella typhimurium*, due to proper processing of human propeptide by murine matrilysin [22].

### Structure of the intestinal epithelium

The mucosal surface of the small intestine consists of crypts of Lieberkühn and villi. Due to the constant shedding of gut epithelial cells in the lumen the whole epithelium renews once in four-five days, except for Paneth cells, which live approximately 70 days. Intestinal epithelium is derived from multipotent columnar stem cells located at the base of the crypt, which expess LGR5 receptor and other stem cell-specific protein markers [30]. An alternative pool of stem cells is positioned higher in the crypt wall [30-32]. Columnar stem cells give rise to actively proliferating transit amplifying cells differentiating into four major epithelial cell lineages: 1) enterocytes absorbing nutrients; 2) goblet cells producing mucus; 3) enteroendocrine cells secreting hormones in the capillaries of the underlying connective tissue (lamina propria); and 4) Paneth cells secreting enteric alpha defensins, as well as other antimicrobials in the mucous layer. Besides, stem cells generate less documented microfold cells (M-cells) responsible for the uptake of mucosal antigens [33] and the recently described tuft cells secreting endogenous intestinal opioids [34]. Paneth cells protect the adjacent stem cells and the whole gut epithelium from microbial infection and regulate bacterial colonization of the gut [35].

### Functions of enteric alpha defensins

**Figure 1:**
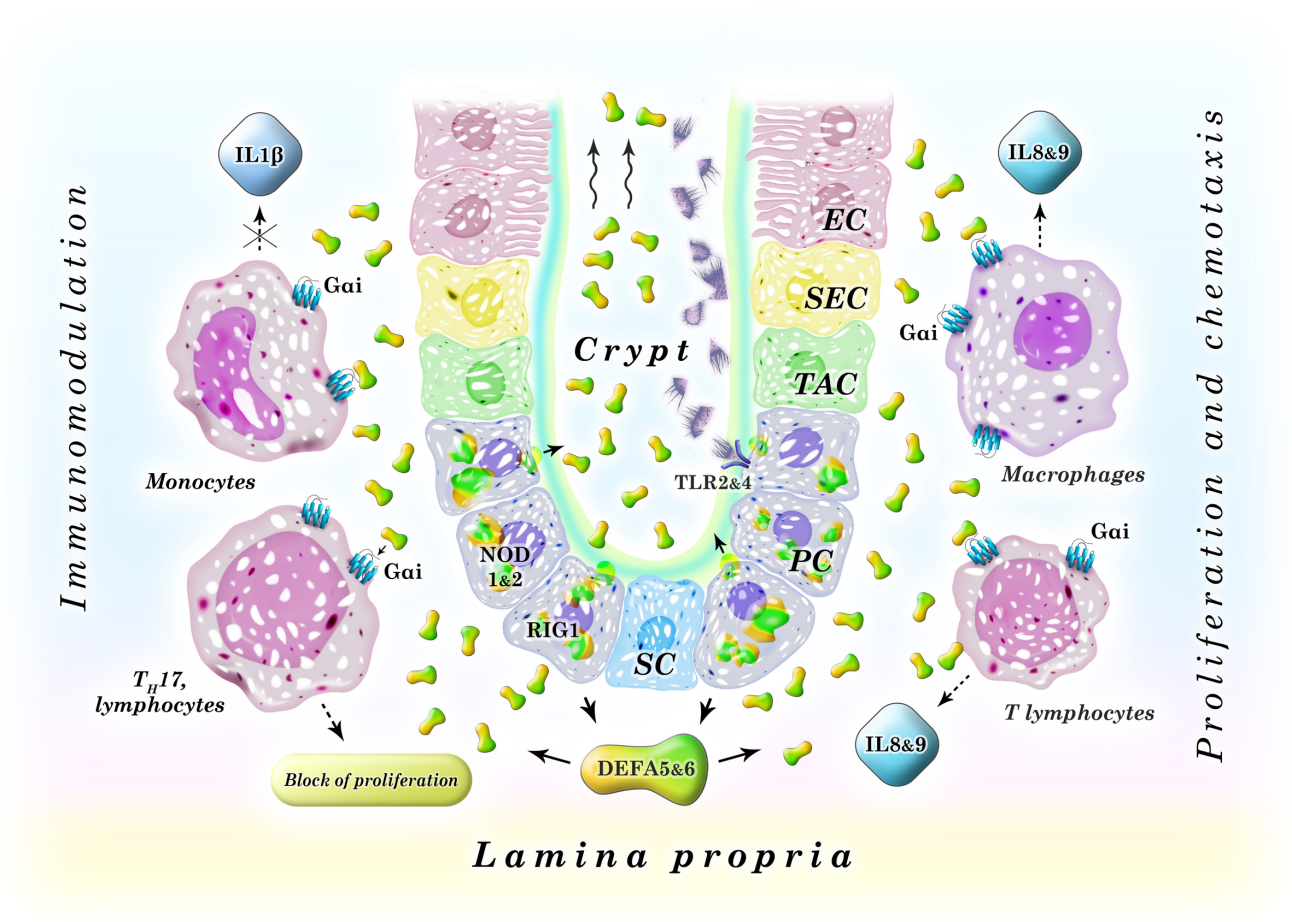
**Schematic diagram of enteric alpha defensins functions in the immune defense of the gut**. Paneth cell receptor activation by microbe's degradation products results in defensins secretion in the lumen (aimed at the regulation of bacterial colonization) and in lamina propria (in order to activate pro- and anti-inflammatory responses of the adaptive immune system cells). SC - stem cells, PC - Paneth cells, TAC - transit amplifying cells, which give rise to two partially differentiated cell lineages: progenitors of secreting cells (SEC) and progenitors of enterocytes (EC).

Enteric alpha defensins are the most abundant products secreted by Paneth cells [36]. The main inducers of their secretion are the products of degradation of Gram-positive and Gram-negative bacteria, inhabiting the gut including: muramyl dipeptide, bacterial lipopolysaccharide, flagellin, lipid A, and unmethylayed CpG sequences in bacterial DNA. Their presence in the mucous layer of the intestinal epithelium is constantly monitored by the three receptor types that are expressed in Paneth cells and enterocytes: 1) toll-like receptors (TLRs, including the most abundant cell surface receptors 2 and 4); 2) cytoplasmic nucleotide oligomerization domain-like receptors (NLRs, in particular NOD1 and 2); and 3) retinoic acid inducible gene 1-like receptors (RLRs, including the most abundant receptor RIG1) (Figure 1) [37]. Receptor signals transmitted by the MAP kinase signaling pathway induce translocation to the nucleus of transcription factors, which initiate transcription of genes that are involved in functioning of the innate and adaptive immune systems and stimulation of inflammation, wound healing, and angiogenesis in the adjacent connective tissues [38-40].

Detection of microbial presence in the mucous layer of the gut results in massive release of enteric alpha defensins by Paneth cells that leads to an enhanced killing of microbes in the lumen. Simultaneous secretion of alpha defensins in lamina propria [12] triggers two opposite defense mechanisms of pro- and anti-inflammatory response. According to the first mechanism, human enteric alpha defensins bind to as yet unidentified Gαi-protein coupled receptors located on the surface of macrophages and T lymphocytes of lamina propria (Figure 1) [41-43], which leads to proliferation of T lymphocytes and their chemotaxis to the site of inflammation [44]. Moreover, it was shown that murine defensins 2 and 3 (as well as beta defensins) may induce reversible formation of ion channels on apical membranes of undifferentiated Cl^- ^secreting cells of the crypt. This results in salt and water secretion, causing the crypt lumen to be flushed after Paneth cell discharge [45].

At the same time enteric alpha defensins moderate intestinal anti-iflammatory response, as a consequence of suppression of IL1 beta release from bacterial lipopolysaccharide-activated monocytes [46] and inhibition of interleukin 17-producing T helper cell proliferation in lamina propria (Figure 1) [36]. In order to avoid the immune response, microbes inject effector proteins into intestinal epithelial cells that either block their immune and inflammatory response (mostly due to inactivation of transcription factors NF-κB or AP-1) or reprogram signaling pathways sensing and killing bacteria [47,48]. Besides, microbes are able to block expression of enteric alpha defensins and other antimicrobial substances by Paneth cells using as yet unknown mechanisms [49].

### Enteric defensins in pathology

Changes in secretion of enteric alpha defensins have been registered in the intestinal epithelium of patients with inflammatory bowel disease (IBD) - a large group of inflammatory illnesses of the gastrointestinal tract, which are caused by immune system over-activation due to a loss of tolerance to gut microflora [50-52]. Two major types of IBD are Crohn's disease (major location - terminal ileum) and ulcerative colitis (predominant lesions in colon and rectum). Susceptibility to Crohn's ileitis in European and North American populations is most strongly associated with mutations in the gene encoding cytoplasmic protein NOD2 - bacterial muramyl dipeptide receptor from NLR family, which is essential for activation of enteric alpha defensins secretion by Paneth cells (Figure 1) [53]. Accordingly, mutations in NOD2 encoding gene (most frequently a frame shift Leu1007fsX1008, caused by single nucleotide insertion 3020insC or "snp13") lead to a decreased level of enteric alpha defensins in the intestinal mucosal extracts [54]. Analysis of Nod2-deficient mice confirmed decreased secretion of alpha defensins 1-6 by Paneth cells and demonstrated inability of such mice to develop intestinal inflammation, which results in their susceptibility to bacterial infection by oral administration [55]. Part of the cases of Crohn's ileitis is independent of NOD2 genotype and is linked to changes in the WNT pathway, mostly due to a decreased activity of transcription factor TCF4 that binds to enteric defensins gene promoters [56].

The second major type of IBD - ulcerative colitis is associated with the infection of gastrointestinal tract by bacterium *Helicobacter pylori *[57]. Subsequent over-activation of the intestinal immune system results in elevated secretion of enteric alpha defensins (as well as TNF alpha and IL1 beta) suggesting that *Helicobacter *is able to avoid the innate and adaptive immune response in human intestine by as yet unknown mechanisms [58].

Similar to ulcerative colitis, 15% of gastrointestinal malignancies arise as a consequence of chronic microbial infections, as demonstrated by the raised chance of hepatocellular carcinoma in patients with chronic hepatitis, association between *Helycobacter pylori *infection and higher gastric cancer risk, and increased chance of colon cancer in patients with inflammatory bowel disease [59]. At initial stages of colon carcinogenesis, mutations in intestinal epithelial cells lead to constitutive activation of the Wnt pathway in early adenoma cells, which simultaneously follow differentiation programs of progenitor and Paneth cells [60]. This results in 60 fold increase of DEFA5 and DEFA6 production by tumors, as compared to normal colonic epithelium [61,62]. Besides, both early adenomas and adenocarcinomas acquire the ability to secrete these proteins into the bloodstream. Thus, enteric alpha defensins are promising markers for early diagnosis of colon cancer provided that test sensitivity is sufficient for their robust detection in sera.

## Conclusion

Enteric alpha defensins play an important role in regulation of bacterial colonization of the gut, as well as in activation of pro- and anti-inflammatory response of the adaptive immune system cells in lamina propria. Further studies of the defensins functions in norm and pathology can provide important clues for the development of new tools for diagnosis and treatment of gastrointestinal cancers and most widespread inflammatory illnesses of the small and large intestine.

## Competing interests

The authors declare that they have no competing interests.

## Authors' contributions

NA drafted the manuscript. YA compiled the reference list. IG performed proofreading. GS designed the figure. Y contributed to the description of defensins functions. SF participated in the design and coordination. All authors read and approved the final manuscript.
